# Quantitative Proteomic Analysis of Serum Proteins from Oral Cancer Patients: Comparison of Two Analytical Methods

**DOI:** 10.3390/ijms150814386

**Published:** 2014-08-18

**Authors:** Yan Yang, Junwei Huang, Bahareh Rabii, Ramin Rabii, Shen Hu

**Affiliations:** 1 Department of Stomatology, Zhongnan Hospital, Wuhan University, Wuhan 430071, China; E-Mail: yangfionayan@gmail.com; 2 School of Dentistry, University of California, Los Angeles, CA 90095, USA; E-Mails: huangjunwei007@gmail.com (J.H.); baharehrabii@gmail.com (B.R.); raminrabii@gmail.com (R.R.); 3 Jonsson Comprehensive Cancer Center, University of California, Los Angeles, CA 90095, USA

**Keywords:** serum proteomics, oral squamous cell carcinoma, mass spectrometry, stable isotope labeling, protein biomarker

## Abstract

Serum proteomic analysis can be a valuable approach for the discovery of protein biomarkers for early detection or monitoring of a disease. In this study, two analytical methods were compared for quantification of serum proteins in patients with oral cancer. In the first approach, we quantified serum proteins between oral squamous cell carcinoma (OSCC) and healthy control subjects by performing in-solution digestion of serum proteins, isobaric tags for relative and absolute quantification (iTRAQ) labeling of the resulting peptides, strong cation exchange (SCX) fractionation of labeled peptides and finally capillary liquid chromatography with tandem mass spectrometry (LC-MS/MS) analysis of the peptides. In the second approach, we first separated serum proteins with SDS-PAGE. The gel-separated proteins were then digested with trypsin and the resulting peptides were labeled with iTRAQ and analyzed with LC-MS/MS for protein quantification. A total of 319 serum proteins were quantified with the first proteomic approach whereas a total of 281 proteins were quantified by the second proteomic approach. Most of the proteins were identified and quantified by both approaches, suggesting that these methods are similarly effective for serum proteome analysis. This study provides compelling evidence that quantitative serum proteomic analysis of OSCC is a valuable approach for identifying differentially expressed proteins in cancer patients’ circulation systems that may be used as potential biomarkers for disease detection. Further validation in large oral cancer patient populations may lead to a simple and low invasive clinical tool for OSCC diagnosis or monitoring.

## 1. Introduction

Oral cancer is a subgroup of head and neck cancer that affects many regions of the oral cavity, such as the lips, tongue, salivary glands, and gums. It is considered as the sixth most common cancer, accounting for nearly 3% of the total cancer burden and results in 128,000 annual deaths globally [1,2]. The most common type of oral cancer is oral squamous cell carcinoma (OSCC), which makes up 90% of all oral cancer cases. Patients with OSCC are often diagnosed at a late stage, and there is a high recurrence rate after treatment, especially in those with neck lymph node metastasis [3]. Despite clinical and treatment advances, the overall 5-year survival rates for oral cancer have remained low and stagnant during the past few decades [4,5].

Although OSCC is commonly diagnosed through oral examination followed by histopathology and computed tomography/positron emission tomography scanning, there has been continuous interest in developing serum protein biomarkers to aid the diagnosis of the disease. Tumor antigens are well received as promising diagnostic biomarkers for human cancers, including OSCC. A number of studies were performed to investigate the clinical utility of tumor antigens, such as carcinoembryonic antigen (CEA), CA-50, CA19-9 and squamous cell carcinoma antigen (SCCA), for OSCC detection [6,7,8,9]. Serum SCCA appeared to be more sensitive than the other tumor antigens and was found to be positive in 38.1% and 41.4% of OSCC patients under testing [8,9]. Studies also revealed other potential serum protein biomarkers for OSCC, such as CYFRA 21-1 (cytokeratin 19-fragments), tumor polypeptide antigen (TPA) and insulin-like growth factor binding protein 3 [10,11]. CYFRA 21-1 has been demonstrated as a promising biomarker for other solid tumors, whereas TPA is a serine protease found in rapidly growing tissue due to its role in forming intermediate filaments of the cellular cytoskeleton, making it a promising candidate for cancer detection. Both CYFRA 21-1 and TPA levels were found to be significantly higher in OSCC patients than healthy controls and benign tumor patients, and they were both reduced in patients 2–3 weeks after surgical resection of their OSCC lesions [11]. Although these potential serum protein biomarkers need to be further validated in large clinical trials, the potential of testing serum protein biomarkers as a simple diagnostic tool for oral/head and neck cancer has been well demonstrated in the previously published studies.

The purpose of our study is to demonstrate and compare two proteomics approaches for quantitative analysis of serum proteins from OSCC patients. The quantitative proteomic analysis was based on isobaric tags for relative and absolute quantification (iTRAQ) and liquid chromatography with tandem mass spectrometry (LC-MS/MS). iTRAQ employs a 4-plex or 8-plex set of amine reactive isobaric tags to derivatize peptides at the N-terminus and lysine side chains. In MS, the same peptides labeled with any of the isotopic tags are indistinguishable (isobaric). Upon fragmentation in MS/MS, signature reporter ions (e.g., *m*/*z* from 114–117) are produced, providing quantitative information for the peptides originated from different protein samples [12]. Using similar quantitative proteomics approaches, we have previously identified serum protein biomarkers for classification of oral cancer patients with lymph node metastasis [13] and revealed that the cAMP response element-binding protein 1 (CREB1) pathway is activated in oral cancer stem-like cells [14]. In the present study, two quantitative proteomics approaches were compared for analysis of serum proteins of oral cancer patients and used to identify differentially expressed serum proteins between OSCC and matched healthy control subjects that might be used as candidate biomarkers for further validation.

## 2. Results and Discussion

**Figure 1:**
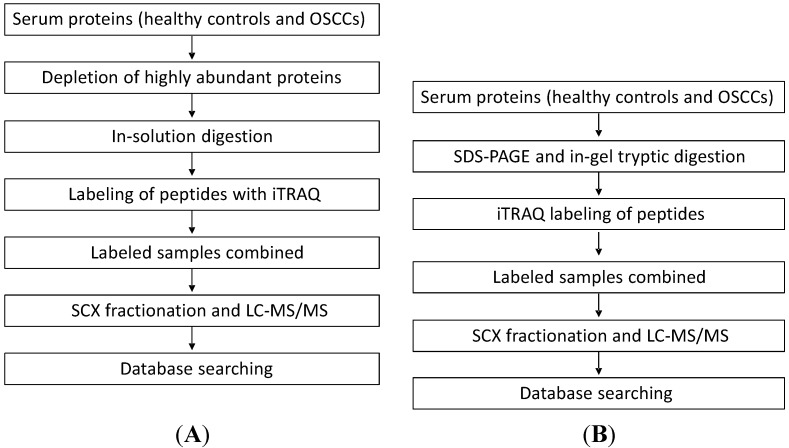
The workflow for the two proteomic approaches used to quantify serum proteins from patients with oral squamous cell carcinoma (OSCC). (**A**) In the first approach, we quantified serum proteins between OSCC and healthy control subjects by performing in-solution digestion of serum proteins, isobaric tags for relative and absolute quantification (iTRAQ) labeling of the resulting peptides, SCX (strong cation exchange) separation of labeled peptides and finally capillary LC with MS/MS analysis of the peptides; and (**B**) In the second approach, we first separated serum proteins with SDS-PAGE. The gel-separated proteins were then digested with trypsin and the resulting peptides were labeled with iTRAQ and analyzed with LC-MS/MS for protein quantification.

Two analytical methods were compared in this study for the discovery of putative serum protein biomarkers of oral cancer. In the first approach, we quantified serum proteins between OSCC and healthy control subjects by performing in-solution digestion of serum proteins, iTRAQ labeling of the resulting peptides, strong cation exchange (SCX) fractionation of labeled peptides and finally capillary LC with MS/MS analysis of the peptides. In the second approach, we first separated serum proteins with SDS-PAGE. The gel-separated proteins were then digested with trypsin, and the resulting peptides were labeled with iTRAQ and analyzed with LC-MS/MS for protein quantification (Figure 1). 

**Figure 2:**
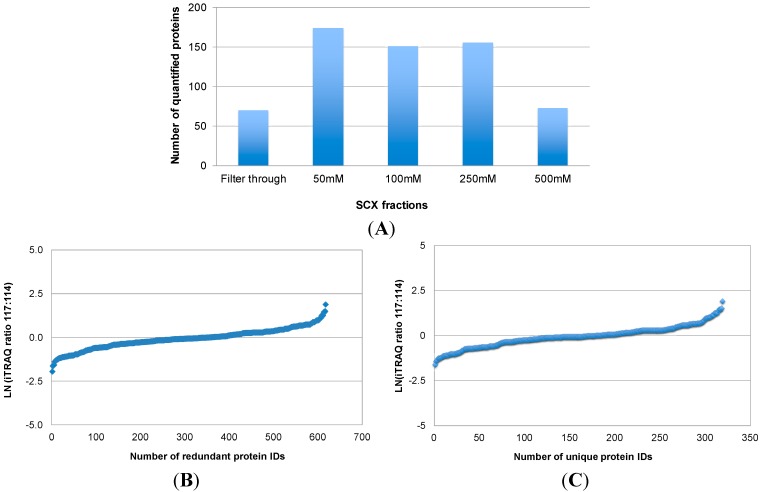
Quantitative proteomic analysis of serum proteins from OSCC patients with Approach 1. In Approach 1, both OSCC and control protein samples were digested in solution, and the resulting peptides were labeled with iTRAQ tags, respectively. Afterwards, the labeled peptides were combined and fractionated with SCX at four different salt concentrations (50, 100, 250, and 500 mM sodium chloride). The filter-through and four SCX fractions were then analyzed by reversed phase LC with MS/MS. The number of proteins quantified in each SCX fractions is shown in (**A**); and their relative levels between OSCC and healthy control subjects are presented in (**B**); The total number of unique proteins quantified by this approach and their relative levels between OSCC and healthy control subjects are shown in (**C**).

Figure 2 illustrates the number of proteins that were quantified by combining iTRAQ with SCX pre-fractionation and LC-MS/MS analysis of labeled peptides (Approach 1). In total, 617 (redundant) protein IDs were obtained from the LC-MS/MS analysis of five SCX fractions (Figure 2A,B), which corresponded to 319 unique proteins. The relative levels of the 319 proteins between OSCC and healthy control subjects are shown in Figure 2B. Quantification of these proteins was based on one or more iTRAQ-labeled peptides from each protein. A major obstacle to serum proteome analysis is the predominance of highly abundant proteins such as albumins, immunoglobulins, alpha-1-antitrypsin, haptoglobin, and their isoforms and fragments. Depletion of these proteins in serum samples is desired for an in-depth proteomic analysis. Immunoaffinity depletion using multiple affinity removal columns is effective because it can simultaneously remove multiple abundant proteins, with minimal carryover and high specificity. Immunodepletion can also be performed with columns packed with antibody-coated microbeads. In our study, we used the IgY-12 SC spin columns, in which affinity-purified anti-IgY antibodies are covalently conjugated through their Fc portion to 60 µm polymeric microbeads, to deplete highly abundant serum proteins prior to quantitative MS analysis. This affinity column is effective to deplete 90%–99% of 12 abundant serum proteins including albumin, IgG, transferrin, fibrinogen, IgA, alpha-2-macroglobulin, IgM, alpha-1-antitrypsin, haptoglobin, alpha-1-acid glycoprotein, apolipoprotein A-I, and apolipoprotein A-II. However, there are two remaining issues with this affinity depletion approach. First, it removes a good portion, but not all of the highly abundant proteins. Most of the 12 abundant proteins were found to be present in the depleted samples, interfering with downstream analysis. The amount of remaining high abundant proteins may vary from sample to sample. This would affect protein assays of the depleted samples, leading to inaccurate iTRAQ quantification. Second, there has been concern regarding whether low-abundance proteins are removed along with high-abundance proteins such as albumin and immunoglobulins via non-specific binding. A possible approach to address this problem is to disrupt the binding of other serum proteins to the carrier proteins albumin/immunoglobulin. For instance, partly denaturing conditions by adding 5%–20% acetonitrile may disrupt the binding, resulting in an increased number of detected proteins in comparison with native conditions [15]. Therefore, in the first approach, we first used 10% acetonitrile to break down the binding between proteins, and then we used immunoaffinity depletion spin columns to remove the highly abundant proteins from serum samples prior to in-solution digestion and iTRAQ labeling.

**Figure 3:**
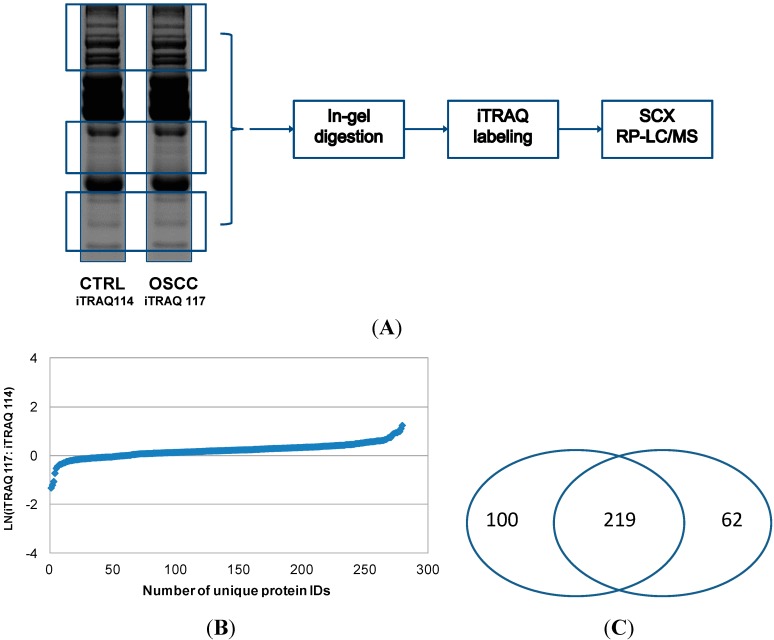
Quantitative proteomic analysis of serum proteins from OSCC patients with Approach 2. In Approach 2, both OSCC and control protein samples were initially separated with SDS-PAGE. Highly abundant proteins within 50–70 kDa and around 28 kDa were avoided, and the rest of proteins were excised for in-gel tryptic digestion (**A**); The resulting peptides were then labeled with iTRAQ tags, respectively, combined and fractionated with SCX. Afterwards, the SCX fractions were analyzed by reversed phase LC (RP-LC) with MS/MS. The number of proteins quantified by Approach 2 and their relative levels between OSCC and healthy control subjects are presented in (**B**); The pie chart shown in (**C**) indicates that most of the proteins are identified and quantified by both analytical approaches.

We also tested another approach for quantitative analysis of serum proteins by combining SDS-PAGE separation of proteins, in-gel digestion and iTRAQ labeling of the resulting peptides, and SCX pre-fractionation with LC-MS/MS of iTRAQ-labeled peptides (Approach 2). Using this method, 281 proteins were quantified, and their relative levels between OSCC and healthy control subjects are shown in Figure 3. In the second approach, rather than depleting highly abundant proteins with affinity columns, we used SDS-PAGE to separate the serum proteins and relatively low abundant protein bands were excised for in-gel tryptic digestion and iTRAQ labeling of the resulting peptides. This approach avoided the most abundant proteins at 55–70 kDa (e.g., serum albumin) and ~28 kDa (e.g., IgG light chain), but those low abundant proteins around 28 kDa or within 50–70 kDa might be lost during the process. Approach 1 represents fractionation at peptide level whereas Approach 2 represents fractionation at protein level. Both approaches identified and quantified a number of medium to high abundant proteins in serum samples, and their relative levels between OSCC and control subjects as revealed by both approaches were similar. However, because in-gel digestion was avoided, more proteins were identified and quantified with Approach 1 than Approach 2, especially the proteins of low abundance. In total, our analysis led to the quantitation of 381 serum proteins, including 219 ones that were quantified by both approaches (Figure 3B).

Supplemental Table S1 presents a combinatory list of serum proteins quantified by the two proteomic approaches described above. As for the 219 proteins quantified by both Approaches, the iTRAQ ratios shown in the Table were averaged from the two analyses. Some of the differentially expressed proteins between OSCC and control subjects may be further validated as potential biomarkers for OSCC detection. Currently OSCC is diagnosed by an oral examination carried out by a physician. If there are any signs or suspicions such as white or red patches, sores, lumps, and ulcers, a small biopsy is performed and sent to a pathologist for histopathological diagnosis and staging. X-rays and computed tomography scans may also be performed to determine if the cancer has spread. The overall five-year survival rate for OSCC is approximately 30% to 40%. Such high morbidity rates can be attributed to poor diagnosis of OSCC. Methodological inconsistencies and lack of principles have led to subjective and unreliable results. As a consequence, OSCC is not diagnosed or treated until the late stages of the disease and has led to high reoccurrence rates. About one in four persons with oral cancer die because of delayed diagnosis or treatment. If OSCC is found early, before the cancer has metastasized, the five-year survival rate is nearly 90%. Recently more advanced detection methods have been developed for visual aid such as *in vivo* optical spectroscopy, tissue staining with toluidine blue, and exfoliated cytology using OralCDx brush biopsy (CDx Diagnostics, Suffern, NY, USA). Despite these advances, there has been little change in early OSCC detection and monitoring. The overall five-year survival rate for OSCC is among one of the lowest of all cancer types for the past few decades.

## 3. Experimental Procedures

### 3.1. Patients and Serum Samples

Serum samples were collected from oral cancer patients and healthy control subjects at the University of California-Los Angeles (UCLA) Medical Center as described previously [16]. All participants provided consent according to the UCLA institutional review board (IRB) approval. The study was conducted in accordance with the Declaration of Helsinki, and the protocol was approved by the UCLA IRB committee. The patients did not receive treatment in the form of chemotherapy, radiotherapy, surgery or alternative medicine prior to sample collection. The total protein concentration of serum samples was measured using the 2-D Quant Kit (GE Healthcare, Piscataway, NJ, USA).

### 3.2. Depletion of High-Abundance Proteins in Serum Samples

To identify protein candidates at differential levels between OSCC and healthy control subjects, we prepared equally pooled serum samples from healthy controls (*n* = 6) or OSCCs (*n* = 6), for quantitative proteomic analysis. These patient and control subjects were matched in terms of gender (*p* = 1.0) and ethnicity (*p* = 1.0). Acetonitrile (final concentration, 5%) was added to each sample as described previously to break down the binding of low-abundance proteins to serum albumin or immunoglobulins [15]. Afterwards, high-abundance proteins present in the pooled serum samples were selectively depleted with IgY-12 SC spin columns (Phenomenex, Torrance, CA, USA) according to the manufacturer’s manual. Afterwards, the protein concentrations of the depleted samples were measured and equal amount of proteins (either OSCC or healthy control) was used for iTRAQ labeling as described below.

### 3.3. iTRAQ Labeling

iTRAQ labeling was performed according to the manufacturer’s instruction manual (Applied Biosystems, Forster City, CA, USA). In brief, after depletion of high-abundance proteins, each pooled sample (100 µg proteins in total) was reduced with 5 mM tris-(2-carboxyethyl) phosphine (TCEP) at 60 °C for one hour and then incubated with 4 mM iodoactamide in the dark at room temperature for 30 min. Afterwards, the samples were digested with 2.5 µg sequencing grade trypsin (Promega, Madison, WI, USA) at 37 °C overnight and then reacted with iTRAQ-114 (control) and iTRAQ-117 (OSCC), respectively. Finally, the labeled peptide samples were combined for 2-D LC separation and MS/MS analysis.

### 3.4. SCX Fractionation and Reversed Phase (RP) LC with MS/MS

The combined iTRAQ-114 and iTRAQ-117 labeled sample was first separated with Vivapure strong cation spin columns (Vivapure S MINI H, Sartorius, Germany) according to the manufacturer’s protocol. Prior to loading into the spin column, the labeled sample was diluted with water (1:8). The eluted fractions from the spin columns at four different salt concentrations (50, 100, 250, 500 mM sodium chloride) were then analyzed using nano-LC (Eksigent Technologies, Dublin, CA, USA) and linear ion trap MS (LTQ XL, Thermo-Fisher Scientific, Waltham, MA, USA). Peptide separation was performed using the PepMap100 C18 column (3 µm, 100 A°, 75 µm I.D., 15-cm length, Dionex, Sunnyvale, CA, USA) with a 300-µm I.D. 3.5-mm-long C18 guard column (Dionex). Peptides were eluted using a linear gradient of 5%–95% LC buffer B (5% H_2_O, 95% ACN containing 0.1% formic acid) over 60 min, followed by isocratic elution at 95% buffer B for 15 min with a flow rate of 400 nL/min across the capillary column. Electrospray (ESI) was performed at 2.0 kV using a PicoTip nanospray emitter (10-µm I.D., New Objective, Woburn, MA, USA).

Peptides were selected for MS/MS using pulsed Q collision induced dissociation (PQD) operating mode with a normalized collision energy setting of 42%. The ion trap was operated in a data-dependent mode, with one MS survey scan (400–1800 *m*/*z*) followed by five MS/MS scans for the five most abundant precursor ions in the MS survey scan. The *m*/*z* values selected for MS/MS were dynamically excluded for 20 s.

The obtained MS/MS spectra were searched against the NCBI non-redundant database using the Sequest search engine. Search parameters were set as follows: taxonomy, Homo sapiens; enzyme, semi-tryptic/tryptic; variable modifications, oxidation (M), phospho (ST), phospho (Y), carbamidomethyl (C), iTRAQ-4plex (K), iTRAQ-4plex (*N*-term), iTRAQ-4plex (Y); peptide mass tolerance, ±1.2 Da; fragment mass tolerance, ±0.6 Da; maximum missed cleavages, 1. Peptides with Xcorr > 2.0 (2+) or Xcorr > 2.5 (3+) were considered as significant match. Confident identification of a protein was based on two or more unique peptides and the false discovery rate was below 2%. Quantification of proteins was based on an average ratio of iTRAQ-labeled peptides from each protein.

### 3.5. SDS-PAGE and In-Gel Tryptic Digestion

Pooled serum samples (either OSCC or healthy control) were first precipitated with cold 10% TCA in acetone. The protein pellets were then dissolved in a buffer containing 7 M urea, 2 M thiourea, 20 mM DTT, 1.2% CHAPS (*w*/*v*), 5% glycerol (*v*/*v*), 10% isopropanol (*v*/*v*), and 0.4% ASB-14 (*w*/*v*). Finally, the serum proteins (30 g, each pooled sample) were separated using 12% NuPAGE gels (Invitrogen, Carlsbad, CA, USA) and stained with Sypro Ruby (Bio-Rad, Fullerton, CA, USA). Selected protein bands (Figure 3A) were excised using the ProteomeWorks™ Spot Cutter (Bio-Rad). In-gel tryptic digestion of the proteins was then performed and the resulting peptides were extracted with 0.1% trifluoroacetic acid (TFA) in 50% acetonitrile. The peptides that resulted from each pooled sample were combined, vacuum dried, and labeled with iTRAQ 114 and 117 for control and OSCC samples, respectively. The labeled samples were finally combined and separated with the Vivapure strong cation spin columns (Vivapure S MINI H, Sartorius, Bohemia, NY, USA), as described above. The eluted fractions from the spin columns at four different salt concentrations (50, 100, 250, 500 mM) were then analyzed using LC with MS/MS.

## 4. Conclusions

In summary, we have demonstrated and compared two analytical methods for quantification of serum proteins in patients with oral cancer based on iTRAQ labeling and LC-MS/MS analysis. These two methods represent fractionation at the peptide or protein level and are similarly effective for serum proteome analysis. As an effort to develop a possibly effective method for diagnosis or prognosis of OSCC, researchers have utilized MS-based proteomics to identify body fluid (e.g., saliva and serum) biomarkers for disease detection/monitoring. We have previously demonstrated serum protein biomarkers may be used to differentiate OSCC patients with lymph node metastasis from those with primary disease [13]. A biomarker that reflects the status of lymph node metastasis is highly desirable to classify patients with OSCC for optimal treatment. Our present study provides compelling evidence that quantitative proteomic analysis of serum samples from OSCC patients may well lead to potential protein biomarkers to facilitate detection of OSCC. Once newly-discovered candidates are validated in large patient populations, they may be used to help detect OSCC or monitor patients (e.g., heavy smokers) who are at high risk for developing OSCC.

## Supplementary Files

Supplementary File 1
